# Oral and Gut Microbial Carbohydrate-Active Enzymes Landscape in Health and Disease

**DOI:** 10.3389/fmicb.2021.653448

**Published:** 2021-12-10

**Authors:** Stanley O. Onyango, John Juma, Kim De Paepe, Tom Van de Wiele

**Affiliations:** ^1^Center for Microbial Ecology and Technology (CMET), Ghent University, Ghent, Belgium; ^2^International Livestock Research Institute (ILRI), Nairobi, Kenya

**Keywords:** microbiota, carbohydrates, diabetes, colorectal cancer, arthritis, glycoside hydrolases, glycosyltransferases, carbohydrate active enzymes (CAZy)

## Abstract

Inter-individual variability in the microbial gene complement encoding for carbohydrate-active enzymes (CAZymes) can profoundly regulate how the host interacts with diverse carbohydrate sources thereby influencing host health. CAZy-typing, characterizing the microbiota-associated CAZyme-coding genes within a host individual, can be a useful tool to predict carbohydrate pools that the host can metabolize, or identify which CAZyme families are underrepresented requiring supplementation via microbiota transplantation or probiotics. CAZy-typing, moreover, provides a novel framework to search for disease biomarkers. As a proof of concept, we used publicly available metagenomes (935) representing 310 type strain bacterial genomes to establish the link between disease status and CAZymes in the oral and gut microbial ecosystem. The abundance and distribution of 220 recovered CAZyme families in saliva and stool samples from patients with colorectal cancer, rheumatoid arthritis, and type 1 diabetes were compared with healthy subjects. Based on the multivariate discriminant analysis, the disease phenotype did not alter the CAZyme profile suggesting a functional conservation in carbohydrate metabolism in a disease state. When disease and healthy CAZyme profiles were contrasted in differential analysis, CAZyme markers that were underrepresented in type 1 diabetes (15), colorectal cancer (12), and rheumatoid arthritis (5) were identified. Of interest, are the glycosyltransferase which can catalyze the synthesis of glycoconjugates including lipopolysaccharides with the potential to trigger inflammation, a common feature in many diseases. Our analysis has also confirmed the expansive carbohydrate metabolism in the gut as evidenced by the overrepresentation of CAZyme families in the gut compared to the oral site. Nevertheless, each site exhibited specific CAZyme markers. Taken together, our analysis provides an insight into the CAZyme landscape in health and disease and has demonstrated the diversity in carbohydrate metabolism in host-microbiota which can be a sound basis for optimizing the selection of pre, pro, and syn-biotic candidate products.

## Introduction

It is well established that diet affects host health on the one hand and plays a critical role in modulating the composition of the host gut microbiota on the other hand. A direct relation between host health and the microbiome has been hypothesized and a dysbiosis, a loss in functional diversity of the microbiome, may co-occur in a disturbed health state (Durazzo et al., 2019).

The link between metabolic activities of microbiota and certain diseases including dental caries has largely been resolved (Touger-Decker and van Loveren, 2003; Obata et al., 2014; Belstrøm et al., 2017). However, for some diseases such as obesity, type 1&2 diabetes, cardiovascular diseases, colorectal cancers, and liver associated diseases, Alzheimer’s among others, evidence of microbiota involvement is not yet clear (Lozupone et al., 2012; Zeller et al., 2014). Mechanistically, how the microbiota attunes host health is still under exploration, nonetheless alterations of the diet regime are proposed in the management of some chronic diseases (Allison, 2017), suggesting a complex three-way interaction between the gut microbiome, host health and diet is in play. Diet can induce a temporary and reversible influence on the microbial community structure (Walker et al., 2011; Li et al., 2017; Leeming et al., 2019) even though some studies have reported that the shift in the microbial community structure could be long term (De Filippo et al., 2010; Wu et al., 2011). In this regard, the expansive enzyme machinery for carbohydrate metabolism might play an important role through the release of health modulating biomolecules such as short-chain fatty acids (butyrate, propionate, and acetate) which are products of carbohydrate fermentation (Koh et al., 2016).

Carbohydrate active enzymes (CAZymes) are encoded by thousands of genes in the microbial genomes as compared to only 17 that are relevant in humans (Cantarel et al., 2012; Kaoutari et al., 2013) revealing that humans do not have such elaborate enzyme machinery for utilizing diverse sources of complex carbohydrates. This necessitates that humans enter into a symbiotic co-metabolism with the microbiota to harvest energy particularly from the indigestible carbohydrates (Soverini et al., 2017).

Microbiota-diet interactions take place in the human gastrointestinal tract representing distinct habitats and niches, including the oral cavity, stomach, small intestine, and large intestine which are largely defined by the fluctuating environmental conditions. The microbiota colonizing these habitats exhibits a wide range of metabolic capacities driven by the substrate [availability, structure, and composition (Payling et al., 2020) and the environmental cues (Di Rienzi and Britton, 2019) and hence can be considered to be site-specific. Nevertheless, some species or strains can also be found across multiple habitats because of their ability to adapt to multiple niches (Di Rienzi and Britton, 2019). For example, it has been revealed that the translocation and colonization of the oral microbiota into the small and large intestine is not aberrant but active suggesting that oral bacteria constantly seed the gut microbiota (Prodan et al., 2019; Schmidt et al., 2019). Although the functional contribution of the translocated microbiota is not well documented, it is plausible that they adapt to their new ecological niche and shape the gut microbial community structure, and can dynamically alter the carbohydrate metabolizing functional niche in the gut environment. Understanding the carbohydrates metabolizing potential of the oral microbiota will provide important insight into their interactions with the gut microbiota and their contribution to host health.

It is worth noting that the CAZyme profile is dynamic and appears to be influenced by not only the available carbohydrates but other factors including non-carbohydrate food sources, mode of delivery, and adult lifestyle (Ye et al., 2019). By extension, inter-individual variability in host CAZymes or the loss of some microbial species with specific or unique CAZymes can profoundly alter how the host interacts with diverse carbohydrate sources (Aakko et al., 2020) thereby altering metabolic functionality of the gut microbiota and potentially also affecting host health. Characterizing the CAZyme complement encoded by the microbial genetic diversity present in an individual host (CAZy-typing) can be useful in predicting carbohydrate pools that the host can metabolize, or which CAZyme families are underrepresented requiring supplementation via microbiota transplantation or probiotics. The usefulness of such predictions is increasingly becoming relevant in clear hypotheses formulation and guided experimentation as has recently been applied in identifying hemicellulose hydrolytic yeast species (Ravn et al., 2021). In order to link disease state with CAZyme profiles, we used publicly available metagenomes to compare the abundance and distribution of CAZymes in saliva and stool samples from healthy subjects and patients suffering from colorectal cancer, rheumatoid arthritis, and type 1 diabetes. The focus was on the glycoside hydrolases (GHs) and polysaccharide lyases (PL) CAZyme families which are responsible for the glycosidic linkage hydrolysis between carbohydrates and between a carbohydrate and a non-carbohydrate moiety, respectively. Furthermore, we have also characterized the glycosylating glycosyltransferases (GTs) CAZymes.

## Methodology

### Dataset and Data Analysis

R version 4.0.3 (2020-10-10; (R Core Team, 2019)) and Excel 2016 were used for data processing and visualization. Statistical significance was tested at α = 0.05.

We explored CAZyme profiles of 470 healthy and diseased subjects represented by intra-individual saliva and stool samples in 935 metagenomes. The 310 metagenome profiled bacterial species from the 935 metagenomes accounted for 99% of classifiable microbial abundance (Schmidt et al., 2019). CAZyme annotation is only possible at the strain level and due to species overlap, reference strains of the 310 metagenomically profiled species were used in the CAZyme annotation. The diseased subjects were from case-controlled studies (Supplementary Table 1) diagnosed with rheumatoid arthritis (Zhang et al., 2015), colorectal cancer (Zeller et al., 2014; Schmidt et al., 2019), and type 1 diabetes (Heintz-Buschart et al., 2016; Schmidt et al., 2019). More details on the subjects are provided in Schmidt et al. (2019). The type strain genomes were downloaded from NCBI and used to characterize the link between disease and CAZymes in the oral and gut ecosystems.

### Carbohydrate-Active Enzymes Annotation in the Gut and Oral Metagenomes

The 310 bacterial genomes were retrieved from the NCBI Genbank FTP site in FASTA format. Carbohydrate-active enzyme (CAZyme) annotation was performed using the standalone version of the dbCAN2 annotation tool (Zhang et al., 2018). To improve the CAZyme prediction accuracy, we used two of the three dbCAN2 integrated tools for automated CAZymes prediction. The DIAMOND tool allows for fast sequence homology searches in the CAZyme database and was set at an E-value < 1e -102 while Hotpep enables the detection of short, conserved motifs in the peptide pattern recognition library. The Hotpep parameters were set at conserved peptide hits > 6 and the sum of conserved peptide frequencies > 2.6. Together, these parameters ensured a maximal best scoring alignment even from novel genomes but with stringency to avoid false positives. First, pre-computed bacterial CAZyme sequences/annotations were fetched from the dbCAN2 meta server^1^ on December 10, 2020. The following sequences/annotations were used^2^ ^3^ ^4^ ^5^ ^6^ ^7^.

For each genomic sequence, the aggregated results file (“overview.txt”) was filtered and only candidates found by at least two tools were kept for downstream analyses. The filtered candidates were grouped by CAZy ID and then a count was obtained for each ID. Subsequently, an aggregated table with CAZy IDs as columns and bacterial taxonomic IDs, taxonomic ranks, and genome size as rows was constructed (Supplementary Table 2). The aggregated CAZyme table was then used to compare CAZyme profiles at the phylum and genus levels.

### Carbohydrate-Active Enzyme Family Normalization and Abundance

All the annotated bacterial CAZyme families: GHs, PL, Carbohydrate-Binding Modules (CBMs), Carbohydrate Esterases (CE), and enzymes that carry out Auxiliary Activity functions (AA) were collated from each genome and retained for analysis. Since the main aim was to characterize catalytic and glycosylation potential of the oral and gut microbiota from healthy and disease subjects, no extensive analysis was carried out on AA, CE, and CBM. The abundance of each CAZyme family in each metagenome was computed as a product of gene copies of each CAZyme family and the relative abundance of the bacterial type strain in each metagenome and the metagenome read depth (total reads). This abundance table (Supplementary Table 2) enabled comparisons between samples (individuals) stratified by health status, as well as body site: gut and oral.


$$i.\;A_{C}^{i\,n\,S}=N_{C}^{i\,n\,S}*R_{S}^{i\,n\,M}*T_{S}$$

$$ii.\;A_{C}^{i\,n\,M}=\frac{\sum_{i=1}^{310}A_{C}^{i\,n\,S}}{310}$$

Where, ${\text{A}}_{C}^{i\,n\,S}$ is CAZyme family abundance of each strain in a metagenome, ***C*** is a CAZyme family, ***S*** is a type strain with a reference genome, ***N*** is CAZyme gene copies of ***C*** in ***S*** genome, ***R*** is the relative abundance of ***S*** in the metagenome, ***M*** is a metagenome, and ***T*** is the bacteria associated reads (total reads) in ***M***. Formal analysis was based on mean abundances of each CAZyme family ${\text{A}}_{C}^{i\,n\,M}$at factor level: disease phenotypes or body sites or at taxon level.

### Carbohydrate-Active Enzyme Profile and Feature Selection Based on Hierarchical Clustering and Sparse Partial Least Square Discriminant Analysis Analysis

A ComplexHeatmap R package (Gu et al., 2016) was used to first visualize in a heatmap phylum-genus specific CAZyme family signatures based on Z score standardization of the mean gene copy numbers of the CAZyme families at the genus level. In addition, a heatmap of CAZyme family abundance in saliva (oral) and fecal (gut) samples of 935 metagenomes derived from healthy controls and patients with type 1 diabetes, colorectal cancer, and rheumatoid arthritis were also constructed based on Z scores of the mean of log_10_-transformed CAZyme family abundances. Hierarchical clustering of the samples (rows) and the CAZymes (columns) was performed based on the Spearman distance metric.

To compare and identify abundant CAZyme signatures of disease status, body site, and phyla, supervised analysis and feature selection with sparse Partial Least Square Discriminant Analysis (sPLS-DA) (Lê Cao et al., 2011) were performed based on mixOmics R package (Rohart et al., 2017). To offset for CAZymes with zero abundances, 1 was added to CAZyme abundances then log_10_ transformed before Z scaling (Bhattacharya et al., 2015). The Z score standardization was achieved by first subtracting the mean of each CAZyme family abundance μ from CAZyme abundances of each metagenome $A_{C}^{i\,n\,M}$ before dividing by the standard deviation$\sigma :z=\frac{\left(A_{C}^{i\,n\,M}-\mu \right)}{\sigma }$. This function scaled the data to a distribution with mean 0 and standard deviation 1. Prior to the sPLS-DA analysis, the model was tuned by setting three parameters; the number of components, the number of CAZymes to retain in each component and the prediction distance to evaluate the classification of prediction performance. In this study we used fivefold cross-validation iterating 50 times and an error rate classification was performed to check the stability of the selected features during the cross-validation process. The lowest error rate indicated the optimal number of features to select on each component in the final model in order to obtain the most discriminative CAZyme for each component. No feature was selected for disease phenotype given the similarity in the CAZyme profile. Finally, the sample and contribution plots with CAZymes with a mean maximum contribution for each sPLS-DA component were plotted.

### Detecting Significantly Differentially Abundant Carbohydrate-Active Enzymes Between Disease Phenotypes and Between Oral and Gut Ecosystems

The CAZyme family abundance in metagenome (Supplementary Table 2) data was used to perform DESeq2 (version DESeq2_1.28.1) analysis (Love et al., 2014). The analysis was conducted to statistically determine the significant differences in CAZyme family abundances between body sites (oral versus gut) and disease phenotypes (colorectal cancer, rheumatoid arthritis, and type 1 diabetes), versus healthy subjects (control). CAZymes with a log twofold change absolute value above 2 and a false discovery rate (FDR) less than 0.01 as determined by Benjamini–Hochberg (BH) correction for multiple hypothesis testing were considered to be differentially abundant (Love et al., 2014). The full model was designed by incorporating the factors of disease, body site, and their interaction. An empirical Bayes shrinkage correction was applied for low counts (Zhu et al., 2018). Wald tests were used for pairwise comparisons by contrasting the disease phenotype and healthy controls on the one hand and the oral and gut body sites on the other hand. *p*-values were adjusted for multiple testing using the Benjamini-Hochberg procedure (Love et al., 2014). EnhancedVolcanoplot package v1.7.16 was used to generate volcano plots showing the -log10 (adjusted p-value) as a function of the log2FoldChange while annotating the most pronounced CAZyme families (Blighe et al., 2018).

## Results

CAZy-typing was performed by annotating CAZymes from 310 reference strains of the metagenome-assembled species that were recovered by Schmidt and colleagues (Schmidt et al., 2019). The species were recovered from 935 saliva and fecal samples of colorectal cancer, type 1 diabetes, rheumatoid arthritis, and healthy subjects (Supplementary Table 1). A total of 220 CAZyme families were identified in the 310 type strains, totaling 17406 copies of CAZyme encoding genes. The CAZyme-coding genes were unequally distributed among the CAZyme families. GHs accounted for the majority (63.8%) of the genes followed by the GTs (25.8%). CE genes represented 6.6%. PL, and CBMs each represented 1.9% while genes encoding for auxiliary functions (AA) were sparse (<0.1%). Together, GH and PL genes associated with the glycosidic-bond cleavage were most abundant at 65% of all the discovered CAZyme-encoding genes. A strong correlation (*r*^2^ = 0.72 between the total CAZyme and the genome size was also established (Supplementary Figure 1).

### Carbohydrate-Active Enzyme Profiling of Symbiotic Oral and Gut Bacteria Reveal a Diverse, Taxon-Specific Symbiotic Microbial Carbohydrate Metabolism

**FIGURE 1:**
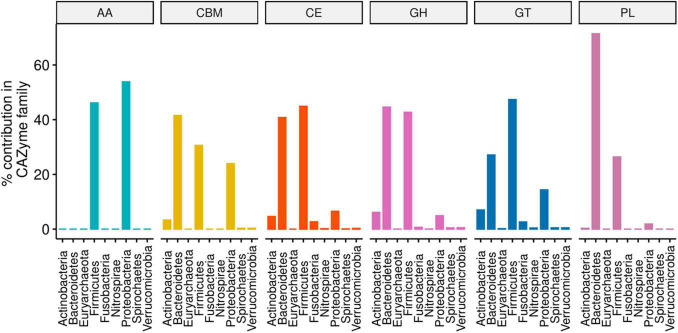
Phylum-level percentage contribution to the abundance of each CAZyme family annotated from 310 type strain bacterial genomes. The proportions were computed on CAZyme family aggregates from a pool of 17406 gene copies obtained from 220 unique CAZyme families.

**FIGURE 2:**
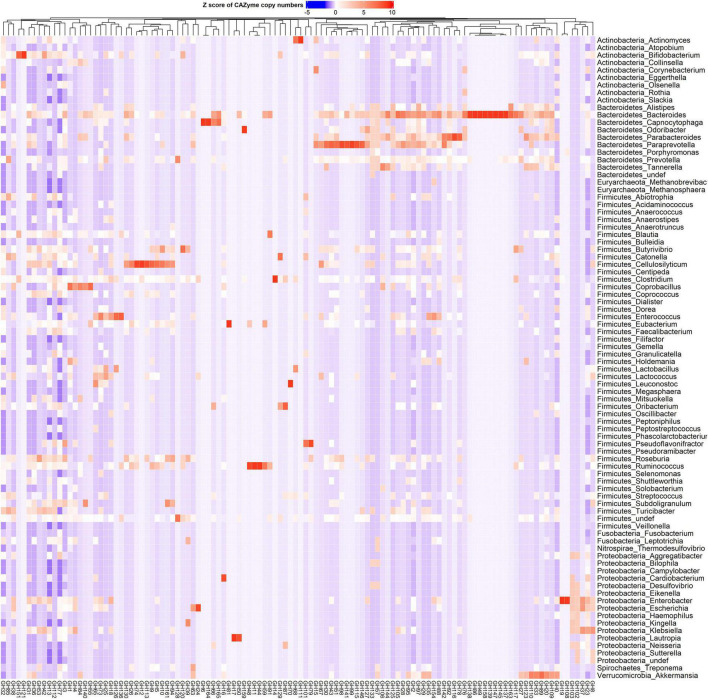
Phylum and genus-specific CAZyme family signatures based on Z score standardization. A heatmap of CAZyme family total gene copy number annotated from 310 reference genomes in 935 metagenomes obtained from the oral (saliva) and gut (fecal) samples of healthy subjects, as well as patients suffering from type 1 diabetes, colorectal cancer, and rheumatoid arthritis. In total, 116 distinct glycosylhydrolases (GHs) families from nine phyla and 86 genera are represented. Each genus displays specific CAZyme enrichments covering a broad carbohydrate range, resulting in a diverse symbiotic microbial metabolism.

**FIGURE 3:**
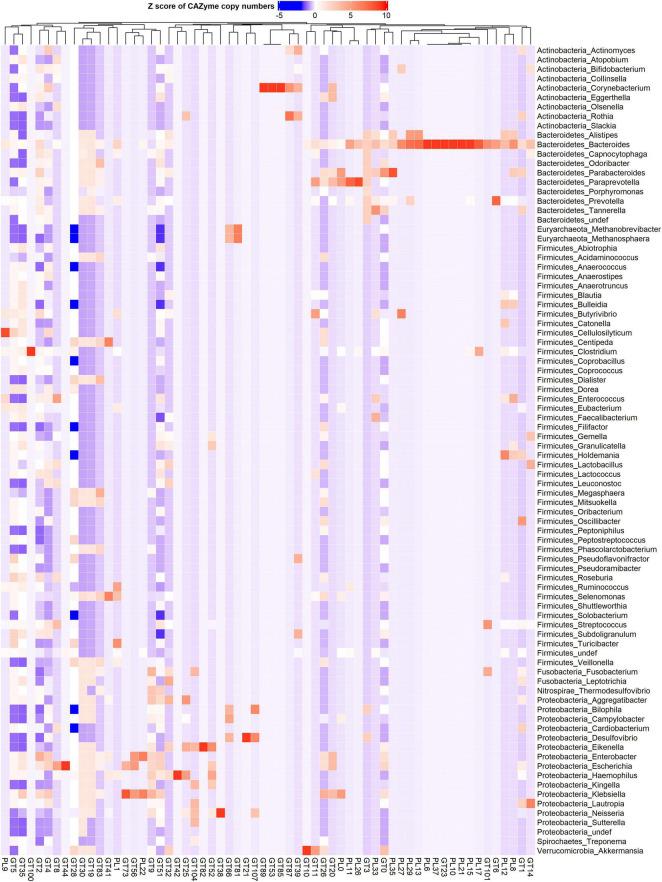
Phylum and genus-specific CAZyme family signatures based on Z score standardization. A heatmap of CAZyme family total gene copy number annotated from 310 reference genomes in 935 metagenomes obtained from the oral (saliva) and gut (fecal) samples of healthy subjects, as well as patients suffering from type 1 diabetes, colorectal cancer, and rheumatoid arthritis. A total of 43 distinct glycosyltransferases (GTs) and 19 polysaccharide lyases (PLs) from nine phyla and 86 genera are represented.

CAZy-typing in all samples (metagenomes) revealed that the total CAZyme gene abundance was largely driven by two Phyla: Firmicutes and Bacteroidota (Figure 1). Moreover, the Firmicutes phylum harbors all the genes encoding CAZymes as opposed to Bacteroidota which is deficient in CAZyme families performing auxiliary functions (AA). GHs and PLs which are associated with the glycosidic bond cleavage were predominantly assigned to the Bacteroidota (44.6% and 71.4%) and Firmicutes (42.7 and 26.4%) (Figure 1), respectively, revealing the importance of Bacteroidota and Firmicutes in carbohydrate metabolism in the host. To better understand how different bacterial clades can contribute to carbohydrate metabolism, we compared CAZyme gene numbers in each of the 310 type strains at the phylum and genus level. A great variation in the number of carbohydrate-metabolizing enzymes (GHs, PLs, and GTs), even within the same genus, was noted (Supplementary Table 2). *Bacteroides*, a dominant genus colonizing the human gut, represented by 19 strains harbored the highest mean number of GHs (155.52 ± 64.14) and PLs (9.52 ± 6.83) (Supplementary Figure 1). By contrast, *Streptococcus*, an abundant genus in the oral cavity, represented by 36 strains harbored fewer GHs (26.33 ± 7.72) and PLs (0.19 ± 0.57) underscoring the variable metabolic capacities of bacterial species in the oral and gut ecosystems. Bacterial specialization in carbohydrate metabolism was evidenced by the hierarchical clustering of CAZymes in the heatmap displaying the CAZyme abundance at the genus level (Figures 2, 3 and Supplementary Figure 2). For example, the *Bacteroides* genus harbors genes encoding for several polysaccharides hydrolyzing enzymes such as PL6 dedicated for animal and alginate glycans, GH163, PL13, PL21, PL29, PL37 targeting animal glycans (chondroitin/heparin), GH137, GH138, GH139, GH145, GH147, PL10, and PL15 dedicated for plant polysaccharides and alginate and GH86, GH117, GH158, PL17, and GH49 for dextran hydrolysis and levan/inulin binding module (CBM66). Overall, the *Bacteroides* genus exhibited an extensive carbohydrate metabolizing potential.

Other genera in the Bacteroidota including *Capnocytophaga* harbor GH6 and GH164 involved in plant polysaccharides hydrolysis, while *Odoribacter* has GH159 associated with plant and animal glycan degradation and levan/inulin binding module (CBM62). *Parabacteroides* harbor GH92, GH116 active on plant polysaccharides, PL35 on animal glycans (chondroitin) and starch (CBM25), and xylan binding modules (CBM13 and CBM9). *Paraprevotella* on the other hand constituted enzymes catalyzing plant polysaccharide breakdown (GH43, GH51, GH115, GH141, GH146, PL11, and PL26), enzymes acting on both plant and animal glycans (GH30 and GH98), and GH99 which is specific for animal glycans and chitin (CBM12) and levan/inulin (CBM62) binding modules.

Also, in the Firmicutes phylum, a large phylogenetic and CAZyme diversity is present (Figures 2, 3 and Supplementary Figure 2). *Cellulosilyticum* demonstrates the largest CAZyme potential including families for plant glycan (GH9, GH10, GH12, GH26, GH74, PL9, and GH5), fungal polysaccharide (GH113) degradation, and CBM25 for starch binding. *Clostridium*, within the same phylum, also harbored GH14 interacting with plant and animal glycans, while *Leuconostoc* GH81 acts on plant polysaccharides. *Eubacterium* on the other hand has GH70 important in dextran utilization enabling the modulation of biofilm development. *Enterococcus* is more enriched in GH126 for starch and glycogen, GH18 and its binding module (CBM12) for chitin, and GH73 for peptidoglycan utilization. *Roseburia* on the other hand has CAZymes adapted to chitobiose/cellobiose (GH94), pectin (GH53) hydrolysis, and also bares rhamnose (CBM67) and starch/glycogen/amylose (CBM42) binding modules. *Coprobacillus* is enriched in CAZyme for fungal polysaccharides (GH55), rhamnogalacturonan (GH140), chondroitin (GH84), and diverse β-glucosides (GH1). Apart from having mannan degrading CAZyme (GH125), *Lactobacillus* can also synthesize fructan from sucrose (GH68) and glycosaminoglycans (GT14). *Ruminococcus* also of the Firmicutes phylum, carries GH11 and GH44 for plant and GH48 for both plant and animal polysaccharide hydrolysis as well as GH1 for chitin/cellulose/xylan binding module (CBM4).

Actinobacteria, in contrast, displayed a more limited CAZyme range (Figures 2, 3 and Supplementary Figure 2). For example, the *Actinomyces* genus is enriched in GH111 tailored toward plant and animal polysaccharide hydrolysis and GH68 which catalyzes the formation of extracellular polysaccharides from sucrose, thereby contributing to biofilm formation. In *Bifidobacterium*, a high abundance of GH151 with fucosidase activity and GH121 engaged in plant and animal polysaccharide hydrolysis was observed. Notably, we also observed that most genera in the Proteobacteria phylum showed specialization in glycosylation through GTs, besides manifesting a carbohydrate hydrolyzing potential. *Delsulfovibrio* can catalyze the biosynthesis of glucosphingolipids via GT21, while *Eikenella, Haemophilus*, *Neisseria*, and *Klebsiella* can synthesize lipopolysaccharides using GT38, GT42, GT73, and GT82. Some other genera such as *Cardiobacterium* (GH16) and *Lautropia* (GH50) harness the potential to hydrolyze marine polysaccharides (agarose).

*Akkermansia*, the only representative of the Verrucomicrobia phylum, exhibited a high abundance of GT10 necessary for fucosylation and can hydrolyze animal (GH89), plant (GH 33 and GH 110), and both plant and animal glycans (GH33).

### CAZy-Typing Reveals That the Carbohydrate-Active Enzyme Profile in Metagenomes Is Largely Determined by Body Site but Not Disease Phenotypes

**FIGURE 4:**
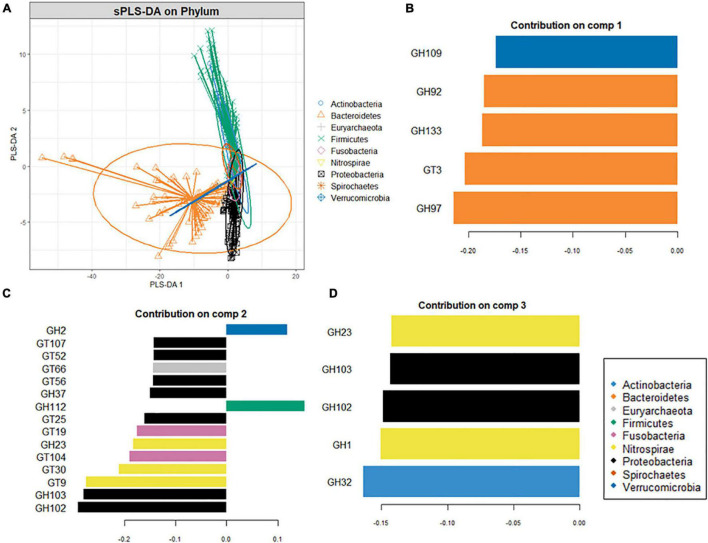
Supervised analysis and feature selection with sparse PLS-DA. **(A)** Sample plots showing clustering of the CAZyme abundance at the phylum level with 95% confidence ellipses. **(B,C)** Loading weights of the most important variables selected on component 1 **(B)**, component 2 **(C)**, and component 3 **(D)**. Fill colors indicate the phylum in which the CAZyme is most abundant. Only the top ten CAZymes are displayed in panel **(B)** while **(A,C)** five CAZymes on which the model was optimal.

Since carbohydrate metabolism requires complex bacterial interactions, the microbial abundance, and community structure of digestive sites can determine the extent of carbohydrate metabolism. Phyla-wise discriminant analysis indicated that the CAZyme features associated with Bacteroidota were well separated from the other phyla (Figure 4A). CAZymes that were most abundant in Bacteroidota (Figure 4B) are involved in the breakdown of animal glycan (GH99 and GH133), plant and animal glycan (GH30 and GH97), and plant glycans (GH92) as well in glycogen synthesis (GT3). Similarly, (Figures 4B,C) CAZymes that break down animal and plant glycans (GH2), animal (GH109), and sucrose/fructan (GH32) were associated with Actinobacteria whereas Proteobacteria (Figures 4C,D), was discriminated by GH37 specifically for trehalose hydrolysis, GH102 and GH103 for peptidoglycans, and as well as CAZymes involved in the biosynthesis of lipopolysaccharides (GT25, GT52, and GT107). Moreover, GH23 (peptidoglycan) and GT9 and GT30 were characteristic of Nitrospirae, while Firmicutes was distinguished by GH112 dedicated to the animal glycan. Fusobacteria was discriminated by GT19 for the synthesis of lipopolysaccharides and GT104 for rhamnosylation of translation elongation factor in bacteria (He et al., 2019).

**FIGURE 5:**
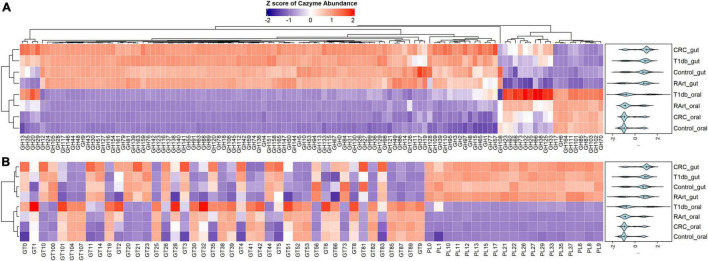
A heatmap of CAZyme family distribution in saliva (oral) and fecal (gut) samples of 935 metagenomes derived from healthy controls and patients with type 1 diabetes, colorectal cancer, and rheumatoid arthritis. A total of 178 distinct CAZymes; 116 glycosylhydrolases (GHs) **(A)**, 19 polysaccharide lyases (PLs), and 43 glycosyltransferases (GTs) **(B)** from nine phyla and 86 genera are represented. The oral and gut ecosystems show distinct profiles revealing a more extensive carbohydrate metabolism in the gut. CRC, colorectal cancer; T1db, type 1 diabetes; RArt, rheumatoid arthritis. The legend shows the Z scores of the mean log-transformed CAZyme family abundance.

**FIGURE 6:**
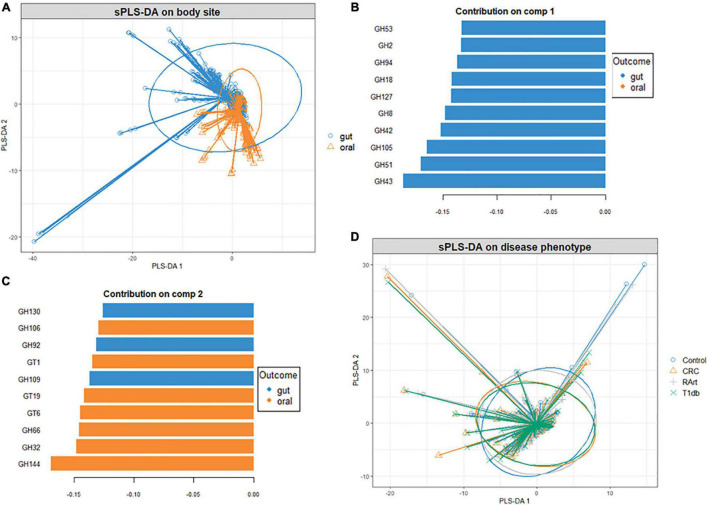
Supervised analysis and feature selection with sparse PLS-DA. Sample plots with 95% confidence ellipses showing clustering of the CAZyme abundance according to body site **(A)**. Loading weights of the most important CAZyme families selected on components 1 **(B)** and 2 **(C)** of the sPLS-DA model with body site as a categorical variable. Fill colors indicate the body site in which the CAZyme is most abundant. Only the top ten CAZymes are displayed. Sample plots with 95% confidence ellipses showing clustering of the CAZyme abundance according to disease condition **(D)**.

A heatmap was constructed to compare CAZymes profiles across the body sites (oral and gut) and disease phenotypes (colorectal cancer, type 1 diabetes, and rheumatoid arthritis) (Figures 5A,B and Supplementary Figure 3) shows that CAZymes from the gut were distinct from the oral samples irrespective of the disease condition. Furthermore, polysaccharide lyases were exclusively found in gut samples. Results of the multivariate sPLS-DA confirmed the distinct composition of CAZymes constituting the gut versus oral habitat (Adonis PERMANOVA test, *p* = 0.01, *r*^2^ = 0.01187) (Figure 6A). Based on the multivariate component analysis, the top ten CAZymes of most importance were selected (Figure 6B) in the first component and comprised of CAZymes abundant in the gut. CAZymes associated with plant cell wall hydrolysis (GH42, GH43 arabinoxylan, GH51 hemicellulose, GH53 pectin), plant and animal glycan degradation (GH2, GH105, GH94 cellobiose, cellodextrin, and chitobiose), animal glycan (GH127), and chitin (GH18) breakdown were observed to be characteristic of the gut ecosystem. GH8, which is known to be important for biofilm synthesis in the oral cavity (Cantarel et al., 2012), was also selected as a determinant of the gut microbial community. In the second component, CAZymes dedicated to glycosylation and breakdown of biofilms were abundant in the oral digestive site (Figure 6C). GH32 which is important for sucrose and fructan hydrolysis, GH66 for dextran utilization, and GH144 which is engaged in glucan hydrolysis were found to characterize the oral cavity. Concerning the disease phenotypes, no distinct stratification (Adonis PERMANOVA test, *p* = 1.00, *r*^2^ = 0.0002) was observed with multivariate sPLS-DA suggesting the overall CAZyme profiles are similar between subjects irrespective of their health status (Figure 6D). Only one component with five CAZymes distinguished the disease status (Supplementary Figure 4). Of the five CAZymes, four were abundant in healthy subjects which comprised of GT56 responsible for the synthesis of enterobacterial common antigen (ECA) trisaccharide units, GH19 responsible for chitin hydrolysis, GH108 engaged in peptidoglycan hydrolysis, and PL22 with pectin metabolizing potential. GH70 with dextran hydrolyzing potential was abundant in rheumatoid arthritis.

### Significant Differences in Carbohydrate-Active Enzyme Abundance Between Body Sites and Disease Phenotypes

**FIGURE 7:**
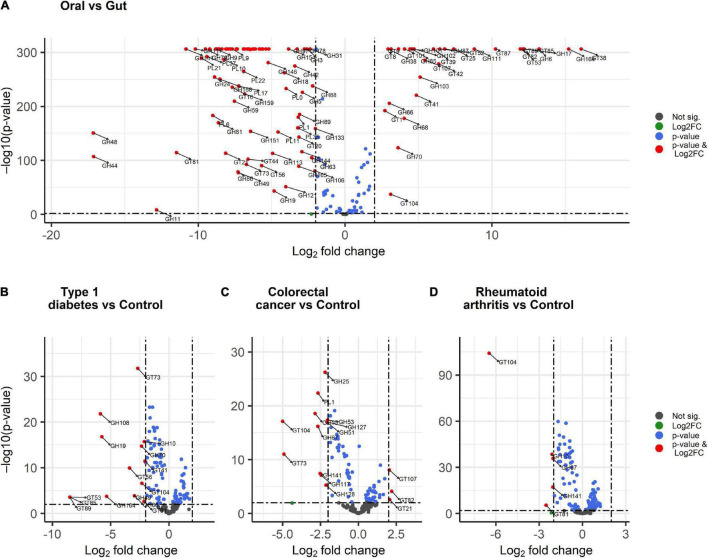
Volcano plots showing differentially abundant CAZymes between body sites and disease conditions. **(A)** oral versus gut, **(B)** colorectal cancer versus control, **(C)** type 1 diabetes versus control, and **(D)** rheumatoid arthritis versus control. The red dots display CAZyme families with both large log2 fold change > 2 or < –2 and a high statistical significance (–log10 of *p*-value, *y*-axis). The blue dots are CAZyme families with points above the horizontal line having *p* < 0.01, the gray dots represent CAZymes having *p* > 0.01 while the green dots are CAZymes with large log2 fold change > 2 or < –2 and *p* > 0.01 and below the horizontal line.

We further explored the statistical significance of CAZymes displaying large differences in abundance (absolute log2FC value exceeding 2) between the gut and oral digestive sites using DESeq2 analysis. Overall, 30 CAZymes, comprised of 17 GTs and 13 GHs had significantly higher abundances in the oral saliva samples (Figure 7A). The differentially abundant GHs in the oral cavity were CAZymes targeting plant (GH164 mannan, GH6 cellulose) and plant and animal glycans (GH17, GH111 starch, and glycogen), while others are dextranases important in biofilm modulation (GH66, GH70, and GH87). The oral site also exhibited the highest glycosylation potential including the synthesis of glucan and lipopolysaccharides (GT9, GT8, GT41, GT42, and GT107) suggesting their importance in dental biofilm. In contrast, 83 CAZymes, including 60 GHs, a few (Leeming et al., 2019) GTs, and 15 PLs were significantly enriched in the gut. Results were consistent with the sPLS-DA (Figure 6A) analysis and 6 CAZymes were identified by both tools: GH94, GH127 catalyzing the hydrolysis of plant and animal glycans (cellulose, agar, and chitin), GH43, GH51, and GH53 associated with plant cell wall digestion and GH18 involved in animal glycan (chitin) breakdown.

A similar DESeq2 approach was used to identify subtle differences between healthy and diseased subjects through pairwise comparisons. We identified 15 CAZyme families that were significantly lower in type 1 diabetes patients (Figure 7B) including GHs acting on peptidoglycan (GH108), chitin (GH19), mannan (GH164) hemicellulose (GH10), and dextran important for glucan synthesis (GH49) as well as GH70 and GH86. Significantly decreased GTs included GT53, GT85, GT89 for the biosynthesis of arabinogalactan in the cell wall, GT56 for the synthesis of enterobacterial common antigen (ECA) trisaccharide units, GT41, GT73, GT81 for lipopolysaccharide synthesis, and GT104 for rhamnosylation. When comparing colorectal cancer and healthy subjects, three CAZymes were significantly differentially enriched (Figure 7C) including GT21 which catalyzes the biosynthesis of glycosphingolipids and GT82 and GT107 engaged in lipopolysaccharide synthesis. In contrast, 12 CAZymes were significantly differentially reduced in colorectal cancer subjects (Figure 7C) including CAZyme families involved in plant and animal polysaccharides hydrolysis PL1 (fucose and cellulose) and GH127 (chitin and cellulose). In addition, glycosylhydrolases specifically acting on plant carbohydrates GH10, GH51 for hemicellulose, GH67 for xylooligosaccharides, GH141, and GH53 for pectin), algal GH117 and GH12, and GH25 involved in the hydrolysis of peptidoglycans were also reduced. Moreover, CAZyme engaged in rhamnosylation (GT104) and synthesis of lipopolysaccharide (GT73) were abundant in control subjects. Finally, rheumatoid arthritis patients demonstrate low abundance in five CAZymes (Figure 7D) involved in the breakdown of hemicelluloses (GH10), cellulose and fucose (GH141), and xylooligosaccharides (GH67). Additionally, GT104 and GT81 involved in lipopolysaccharide synthesis were also reduced in rheumatoid arthritis. Interestingly, GT104 and GH10 were underrepresented in all disease subgroups as compared to the healthy condition. From DESeq2 analysis (Figure 7A), GT104 is more enriched in the oral site and is abundant in *Neisseria, Eikenella*, and *Lautropia* of the Proteobacteria phylum (Figure 3) while GH10 is more enriched in the gut and is abundant in the *Cellulosilyticum* of the Firmicute phylum.

## Discussion

Host and microbiota have both developed CAZymes for the utilization of diverse carbohydrate sources. Although the adult microbiota is relatively stable, a perturbation of the microbiota in a dysbiotic state can lead to an altered CAZyme landscape that can interfere with the carbohydrate metabolism. In this analysis, we have characterized the CAZymes in oral and gut metagenomes from healthy and diseased subjects. We have established that the disease phenotype did not drastically alter the CAZyme gene profile, whereas the gut and oral CAZyme landscape is very distinct and the fecal samples were characterized by more diverse and expansive carbohydrate metabolism potential. We have also identified unique and significant CAZyme signatures based on a pairwise comparison between the healthy controls and disease conditions that are underrepresented in type 1 diabetes (15), colorectal cancer (12), and rheumatoid arthritis (5). These differences almost entirely comprised a significant reduction of certain CAZyme families in diseased states yet for colorectal cancer, CAZyme markers (3) important for lipopolysaccharide synthesis were enriched. Hence our analysis established that even though certain carbohydrate functionality is lost the CAZyme profile is largely conserved, maintaining a great level of carbohydrate metabolic functionality even in certain disease conditions.

Dietary carbohydrates are important biomolecules to both humans and the microbiota since they are the single most efficient energy source as compared to proteins, fats, and lipids. They are broadly classified as simple (mono and disaccharides) and complex carbohydrates which include plant and animal-derived polysaccharides (cellulose, hemicellulose, glycogen, chitin, and starch) (Hölemann and Seeberger, 2004) requiring a broad range of enzymes for their metabolism. The microbiota CAZymes are important for degrading endogenous and exogenous, simple to complex carbohydrates with high specificity (Kaoutari et al., 2013). The specificity requires that host microbiota foster substrate and metabolic cross-feeding within and between phylogenetically diverse communities for efficient carbohydrate utilization (Lozupone et al., 2012; Payling et al., 2020). The diversity in the microbial CAZyme landscape is of critical importance and constitutes a survival mechanism since the microbiota is constantly exposed to a large but fluctuating pool of carbohydrates in the human diet (Di Rienzi and Britton, 2019). Our analysis has indeed demonstrated that most bacteria have developed an expansive carbohydrate metabolizing capacity. For example, *B. thetaiotaomicron* which is a gut bacteria (Xu et al., 2003; Kaoutari et al., 2013) can encode as many as 313 (this analysis) CAZyme families involved in the metabolism of carbohydrates. These includes fucosylated glycans of the gut epithelium, which are particularly important nutrient source when the digestible carbohydrates that require low metabolic cost are limited (Kashyap et al., 2013; Townsend et al., 2019).

The distinct and clear distribution of the CAZyme families between the oral and gut ecosystems reveals the specialization of carbohydrate metabolism in each digestive site, which is largely driven by the carbohydrate availability (Seo et al., 2020) therefore, the CAZyme profile reflects the bacterial community structure and their competitive fitness within the oral and gut sites. Consistent with this study, Cantarel et al. (2012) reported a site-specific CAZyme profile in 500 metagenomes from five body sites with a higher CAZyme abundance in the gut versus the oral cavity. This further confirms the more extensive carbohydrate metabolism that takes place in the gut in comparison to the oral site. Fluctuations and modulation of the microbiota composition and nutrients in these regions appear to be an overall important factor in determining the CAZyme profile.

The short transit time of food in the mouth limits the digestion of diverse complex carbohydrates that humans are constantly exposed to. Our analysis has confirmed that the most abundant CAZymes in the oral cavity are GTs, a group of enzymes that are important in the glycosylation of glycans and aglycons (Tytgat and Lebeer, 2014). They can generate glycoconjugates that can mediate host-microbe interactions including microbial virulence and host immunity (Ovchinnikova et al., 2016). In the oral cavity, GTs catalyze the biosynthesis and secretion of extracellular polysaccharides which are a major component of a mature biofilm (Cugini et al., 2019). Biofilm biosynthesis can also be linked with the presence of sucrose and fructan hydrolases GH32, dextranases GH66, GH77, GH87, glucan endohydrolases GH17, and GH144 in the oral cavity. While our analysis has revealed a more limited carbohydrate degradation potential in saliva samples, the presence of GH6 with cellulolytic activities suggests that complex carbohydrate digestion starts in the mouth and can be attributed to *Capnocytophaga* (Figure 2). This is also in agreement with an earlier study that observed GH6 being abundant in the oral cavity (Cantarel et al., 2012).

Besides alterations in CAZyme profiles according to body sites as a result of nutritional specialization (Cantarel et al., 2012), CAZyme profiles have been demonstrated to change based on geographical locations, ethnicity which is influenced by dietary patterns (Bhattacharya et al., 2015), and age (Ye et al., 2019). The mode of delivery is thought to be the first determinant that can influence the early development of the CAZyme landscape, which can then undergo various transitions from childhood to adulthood when new foods are introduced in the human diet at weaning (Ye et al., 2019). It is unclear whether the early-life seeds set the foundations of a stable host CAZyme profile or if this profile is subjected to drastic changes over time. By interfering with microbiota composition, chronic disease conditions can for instance affect the CAZyme landscape thereby altering the biosynthesis of essential metabolites such as butyrate, an important immunomodulating biomolecule (Koh et al., 2016; Sivaprakasam et al., 2016). Based on sPLS-DA, there was no clear separation between healthy and diseased subjects suggesting that the diseases under investigation did not substantially alter the CAZyme landscape. This can be explained by the shared core microbiota between health and disease (Supplementary Figures 5A,B) suggesting a conserved carbohydrate metabolic functionality. Since the intra-individual microbiota heterogeneity is known to decrease with age (Ye et al., 2019), it is conceivable that the CAZyme profile based on this developed stable microbiota community, undergoes limited perturbation, explaining the similarity of the CAZyme profiles between the diseased and healthy adult subjects observed in our analysis. Therefore, factors that maintain the microbiota homeostatic state would also stabilize an individual’s CAZyme profile and vice versa.

Type 1 Diabetes is a common metabolic disorder with a steadily rising incidence even among children and young adults. This autoimmune-mediated disease develops with a progressive loss of insulin-producing β-cells in the islets of Langerhans in the pancreas (Gülden et al., 2015). Based on microbiota composition, type 1 diabetes is marked by an increase in Bacteroidota, normally dominated by *Bacteroides* in comparison to *Prevotella*, and a low abundance of Firmicutes (Bäckhed et al., 2005; de Goffau et al., 2013). Firmicutes members are associated with high butyrate production (Bäckhed et al., 2005; de Goffau et al., 2013). Foremost butyrate provides energy to the colonocytes as well as regulating the assembly of tight junctions and transepithelial permeability (Bäckhed et al., 2005; de Goffau et al., 2013). Tight junctions restrict the passage of pathogens, microbes, or toxins into the host cells (Groschwitz and Hogan, 2009). Increased gut permeability is thought to precede type 1 diabetes development and has been demonstrated in rat models (Li et al., 2010). Other studies involving *in vitro* and *ex vivo*, suggest that a low butyrate concentration is beneficial and that a high concentration of butyrate could disrupt the mucosal barrier (Peng et al., 2009). Butyrate together with other short-chain fatty acids can directly modulate host immunity. For example, by activating metabolite sensing G protein-coupled receptors (GPR41, GPR43, and GPR109A) in the T lymphocytes can inhibit the deacetylation of histones thereby interfering with the post-translation modification of proteins (Koh et al., 2016; Sivaprakasam et al., 2016). This results in the increase of mucosal Tregs, decreased production of inflammatory cytokines, such as interleukin-10 (IL-10) and interferon-gamma (Durazzo et al., 2019). Tregs play a critical role in the maintenance of immune homeostasis (Atarashi et al., 2011) and it has been shown that short-chain fatty acids can promote its expansion and differentiation (Arpaia et al., 2013). Furthermore, microbial antigens related to butyrate-producing bacteria such as *Clostridium* IV and XIVa can directly induce the Treg in the colon (Atarashi et al., 2011) suggesting the protective effect against this autoimmune disease.

*Bacteroides* members aid the host in digesting otherwise indigestible dietary polysaccharides owing to their expansive CAZyme profile. However, the capacity to degrade even fucosylated glycans (mucin) of the gut lining when dietary fiber is deprived may increase the gut lining permeability, thereby inducing inflammation (Kashyap et al., 2013; Townsend et al., 2019), a condition that can trigger an autoimmune disease. In the present analysis, we have established compromised hydrolysis of chitin, mannan, and hemicellulose due to the underrepresentation of some of the requisite CAZymes (Touger-Decker and van Loveren, 2003) in type 1 diabetes as compared to the control subjects potentially reducing the levels of short-chain fatty acid including butyrate.

Equally important are lactate and acetate-producing bacteria such as *Bifidobacterium* and *Lactobacillus*. Lactate and acetate form the primary substrate for butyrate production. It has further been reported that a low abundance of bifidobacteria, *B. adolescentis*, and *B. pseudocatenulatum*, increases the risk of β-cell autoimmunity (de Goffau et al., 2013).

Similar to type 1 diabetes, rheumatoid arthritis is a chronic autoimmune disease and is characterized by citrullination. Citrullination is a post-translational modification of proteins in which peptidylarginine deiminase substitutes arginine to produce citrulline resulting in immunogenicity (van Venrooij and Pruijn, 2014). The resultant protein may lose structure and function and compromise the structural integrity of cartilage and collagen (György et al., 2006; van Venrooij and Pruijn, 2014). We observed that only a few (5) CAZymes were differentially reduced. Notably, the observed low abundance in GH10, GH67, and GH141 that are involved in the hydrolysis of dietary fibers containing cellulose, xylooligosaccharides, and fucosylated glycans also portends a reduction in capacity to produce short-chain fatty acids. Several studies have reported the protective effect of short-chain fatty acids including butyrate in rheumatoid arthritis (Zhang et al., 2015; Maeda and Takeda, 2019; Rosser et al., 2020). For example, butyrate supplementation reduced the arthritis severity in mice by increasing a serotonin-derived metabolite 5-Hydroxyindole-3-acetic acid which has been shown to suppress the β-cell differentiation (Rosser et al., 2020).

Even though no causal link with host-microbiota has been established, the available evidence supports the hypothesis that dysbiosis in the oral and gut microbiota plays a role in the development of rheumatoid arthritis (Zhang et al., 2015; Maeda and Takeda, 2019). Durholz and others established that a durable Firmicutes-to-Bacteroidota ratio that lasted for over 40 days after the fiber intervention reduced pro-inflammatory cytokines IL-18 while increasing circulating Tregs in rheumatoid arthritis patients (Dürholz et al., 2020) and that a shift of microbial community in favor of Bacteroidota over Firmicutes was associated with low short-chain fatty acids and increased pro-inflammatory response (Dürholz et al., 2020). In agreement, the microbial data used in this analysis revealed that species belonging to the *Bacteroides* including *Bacteroides stercoris*, *Bacteroides* sp. D20, *Bacteroides coprocola*, *Bacteroides caccae*, *Bacteroides vulgatus*, and *Alistipes putredinis* as well as Firmicutes *Ruminococcus bromii* were enriched in the gut of rheumatoid arthritis patients as compared to healthy subjects, while *Prevotella copri* which has been linked with rheumatoid arthritis (Maeda and Takeda, 2019) was reduced (Supplementary Figures 5A,B). Some oral species including *Porphyromonas gingivialis* (Wegner et al., 2010) and *Aggregatibacter actinomycetmcomitans* (Konig et al., 2016) can also promote hypercitrullination of protein linking these oral species with rheumatoid arthritis. Since *Bacteroides* have versatile CAZymes, the enrichment of *Bacteroides* appears to in general conserve the CAZyme profile in rheumatoid arthritis as healthy subjects. Nonetheless, the deficient marker CAZymes in rheumatoid arthritis suggests a compromised short-chain fatty acid production that can favor autoimmunity.

The role of the human microbiome in the development of colorectal cancer has also been supported by the identification of key species that translocate from the oral cavity to the colon (Schmidt et al., 2019). The oral species *Fusobacterium nucleatum* has consistently been implicated in colorectal cancer (Kostic et al., 2013; Rubinstein et al., 2013; Zeller et al., 2014; Schmidt et al., 2019). Other species related to colorectal cancer include *Solobacterium moorei, Porphyromonas asaccharolytica, Parvimonas micra, Peptostreptococcus stomatis, and Parvimonas* spp. (Thomas et al., 2019). Moreover, *Bacteroides fragilis* can induce carcinogenesis in epithelial cells via the production of pro-inflammatory toxins (Sun and Kato, 2016; Chung et al., 2018).

While gut microbiota dysbiosis is thought to be a major driver of colorectal cancer development (Kosumi et al., 2018), robust analysis has provided evidence that though key species are enriched, the gut microbial diversity of colorectal cancer subjects is similar to and in some cases even higher than that of healthy subjects (Thomas et al., 2019). Owing to the conserved diversity, the CAZyme profile and hence carbohydrate metabolism in colorectal cancer subjects is unlikely to be substantially altered by the influx of oral bacteria. Some of the key oral species which were also present in our analysis including *Fusobacteria nucleatum* (Zhang et al., 2015), *Porphyromonas asaccharolytica* (Payling et al., 2020), *Solobacterium moorei* (Wu et al., 2011), *Peptostreptococcus stomatis* (Li et al., 2017), and *Parvimonas micra* (Zeller et al., 2014) have a paltry total number (in parentheses) of total CAZymes in their genomes indicating a lack of robustness in carbohydrate metabolism. As such the subtle changes and influx of oral microbiota associated with colorectal cancer may be sufficient to initiate disease but not exert drastic changes in carbohydrate metabolism, hence conserving the CAZyme landscape. Nevertheless, we have also identified CAZyme markers that are diminished in colorectal cancer yet are important in the utilization of fucosylated glycans, cellulose/hemicellulose, chitin, xylooligosaccharides, pectin, and algal polysaccharides. Potentially, this reduces the generation of short-chain fatty acids such as butyrate which has also been reported to reduce the risk for cancer development (Sun and Kato, 2016). Moreover, we observed an increased abundance in glycosylating CAZymes engaged in the synthesis of glycosphingolipids and lipopolysaccharide in the colorectal cancer samples suggesting their role in cancer pathogenesis.

The potential role of glycosyltransferase in immunomodulation cannot be underestimated. For example, in this analysis, we have observed that most of the Proteobacteria-related species exhibited glycosylation specialization via diverse GTs. For instance, *Eikenella, Haemophilus*, *Neisseria*, and *Klebsiella* (GT42, GT38, GT73, and GT82) can synthesize lipopolysaccharides on bacteria surfaces while *Delsulfovibrio* instead can use GT21 to biosynthesize glycosphingolipids similar to lipopolysaccharides on the surface of bacteria (Kawasaki et al., 1994; Aerts et al., 2019). These capsular polysaccharides and/or lipopolysaccharides molecules are important virulence factors in many bacteria (Weintraub, 2003) and can aggravate epithelial inflammation thereby inducing microbial pathogenesis in many diseases. Thus, in combination with conventional therapies, selectively manipulating microbiota composition to restore the underrepresented CAZymes in the diseased conditions can provide a viable approach for therapy.

## Conclusion

We have confirmed that the gut and oral environment have a distinct CAZyme profile and that the gut microbiome performs the most expansive carbohydrate metabolism. In addition, chronic diseases were found to have a low impact on the CAZyme landscape which is reflected in the conserved CAZyme profiles and suggests functional redundancy of microbiota in the gut and oral ecosystems from the studied disease phenotypes. Despite the absence of major shifts in the CAZyme profile between healthy and disease subjects, differential abundance analysis revealed marker CAZymes compared to the healthy subjects. Further research is still needed to determine the biological significance of the identified CAZyme signatures. The genetic prediction performed in this study cannot establish changes in gene expression, which can determine the enzyme functionality and activity of enzymes and hence the released metabolites resulting from carbohydrate metabolism. Therefore, different disciplines including enzymology, transcriptomics, structural biochemistry, and metabolomics should be combined to decipher how CAZymes sense, metabolize carbohydrates, and impact human health. Despite the challenges, our analysis has given insights into the CAZyme landscape in health and disease and further revealed the diverse metabolic potential of the host microbiota. CAZy-typing can be a very useful tool for clear hypotheses generation and guided experiments with the potential to optimize the selection of pre, pro, and syn-biotic treatment strategies.

## Data Availability Statement

Publicly available datasets were analyzed in this study. This data can be found here: https://doi.org/10.7554/eLife.42693.015 – cohort metadata and microbial reads in metagenomes, https://doi.org/10.7554/eLife.42693.016 – MAGs Tax ID, relative abundances, phylogenetic information.

## Author Contributions

TV: conceptualization, funding acquisition, and writing–review and editing. SO: conceptualization, data curation, formal analysis, writing original draft, and writing–review and editing. KD: methodology and writing–review and editing. JJ: CAZyme annotation and collation. All authors contributed to the article and have given their express approval for the submitted version.

## Conflict of Interest

The authors declare that the research was conducted in the absence of any commercial or financial relationships that could be construed as a potential conflict of interest.

## Publisher’s Note

All claims expressed in this article are solely those of the authors and do not necessarily represent those of their affiliated organizations, or those of the publisher, the editors and the reviewers. Any product that may be evaluated in this article, or claim that may be made by its manufacturer, is not guaranteed or endorsed by the publisher.

## Footnotes

1
http://bcb.unl.edu/dbCAN2/

2
http://bcb.unl.edu/dbCAN2/download/CAZyDB.07312019.fa.nr

3
http://bcb.unl.edu/dbCAN2/download/Databases/dbCAN-HMMdb-V8.txt

4
http://bcb.unl.edu/dbCAN2/download/Databases/tcdb.fa

5
http://bcb.unl.edu/dbCAN2/download/Databases/tf-1.hmm

6
http://bcb.unl.edu/dbCAN2/download/Databases/tf-2.hmm

7
http://bcb.unl.edu/dbCAN2/download/Databases/stp.hmm

## Supplementary Material

Supplementary Figure 1 Correlation between total gene copies of CAZymes per genome and genome size of the 310 type strain recovered from 935 saliva and stool samples of both healthy and diseased subjects.

Supplementary Figure 2 Phylum and genus-specific CAZyme family signatures based on Z score standardization. A heatmap of CAZyme family totals annotated from 310 bacterial genomes in 935 metagenomes obtained from the oral (saliva) and gut (fecal) samples of healthy subjects, as well as patients suffering from type 1 diabetes, colorectal cancer, and rheumatoid arthritis. A total of 27 distinct Carbohydrate-Binding Module (CBM), 13 Carbohydrate Esterase (CE), and 2 CAZymes performing Auxiliary Activity functions (AA) from nine phyla and 86 genera are represented. Each genus displays specific CAZyme enrichments covering a broad carbohydrate range, resulting in a diverse symbiotic microbial metabolism.

Supplementary Figure 3 A heatmap of CAZyme family distribution in saliva (oral) and fecal (gut) samples of 935 metagenomes derived from healthy controls and patients with type 1 diabetes, colorectal cancer, and rheumatoid arthritis. A total of 27 distinct Carbohydrate-Binding Module (CBM), 13 Carbohydrate Esterase (CE), and 2 CAZymes performing Auxiliary Activity functions (AA) from nine phyla and 86 genera are represented. The oral and gut ecosystems show distinct profiles revealing a more extensive carbohydrate metabolism in the gut. CRC, colorectal cancer; T1db, type 1 diabetes; RArt, rheumatoid arthritis. The legend shows the Z scores of the mean log-transformed CAZyme family abundance.

Supplementary Figure 4 Supervised analysis and feature selection with sparse PLS-DA. With the disease status as a categorical variable, the loading weight of the most important CAZyme families selected on component 1 on which the sPLS-DA model was optimal was drawn. Fill colors indicate the disease status in which the CAZyme is most abundant.

Supplementary Figure 5 Relative abundance of the gut and oral microbial community faceted by disease phenotype at the **(A)** genus and **(B)** species level. The abbreviations are disease phenotypes, respectively; CRC, colorectal cancer; RArt, rheumatoid arthritis; T1db, type 1 diabetes. Relative abundance data are adapted from Schmidt et al. (2019).

Supplementary Table 1 Sample metagenome mean read depth and number of samples per body site and disease phenotypes.

Supplementary Table 2 CAZyme family gene copies per genome of the 310 strains used in this study together with their taxonomic ranks, the genome size (Sheet 1). Sheet 2 contains the CAZyme family abundances aggregated per metagenome and metagenome metadata.
