# Supplementary figures and images for: Oral and Gut Microbial Carbohydrate-Active Enzymes Landscape in Health and Disease
Source: Front Microbiol. 2021 Dec 10;12:653448. doi: 10.3389/fmicb.2021.653448 (PMC8702856; doi:10.3389/fmicb.2021.653448)

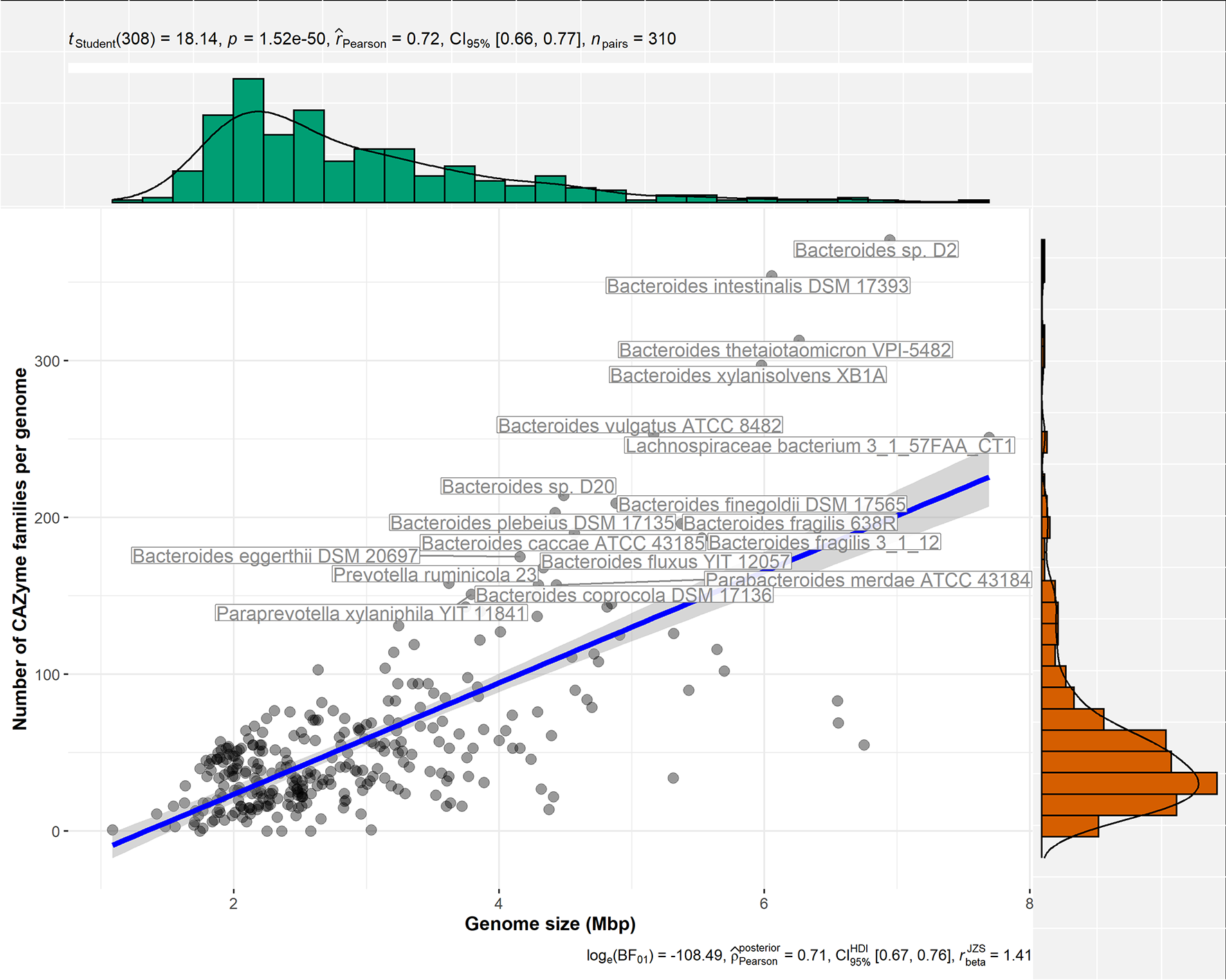

Supplement: Supplementary Figure 1 — Correlation between total gene copies of CAZymes per genome and genome size of the 310 type strain recovered from 935 saliva and stool samples of both healthy and diseased subjects. [file Image_1.TIFF]

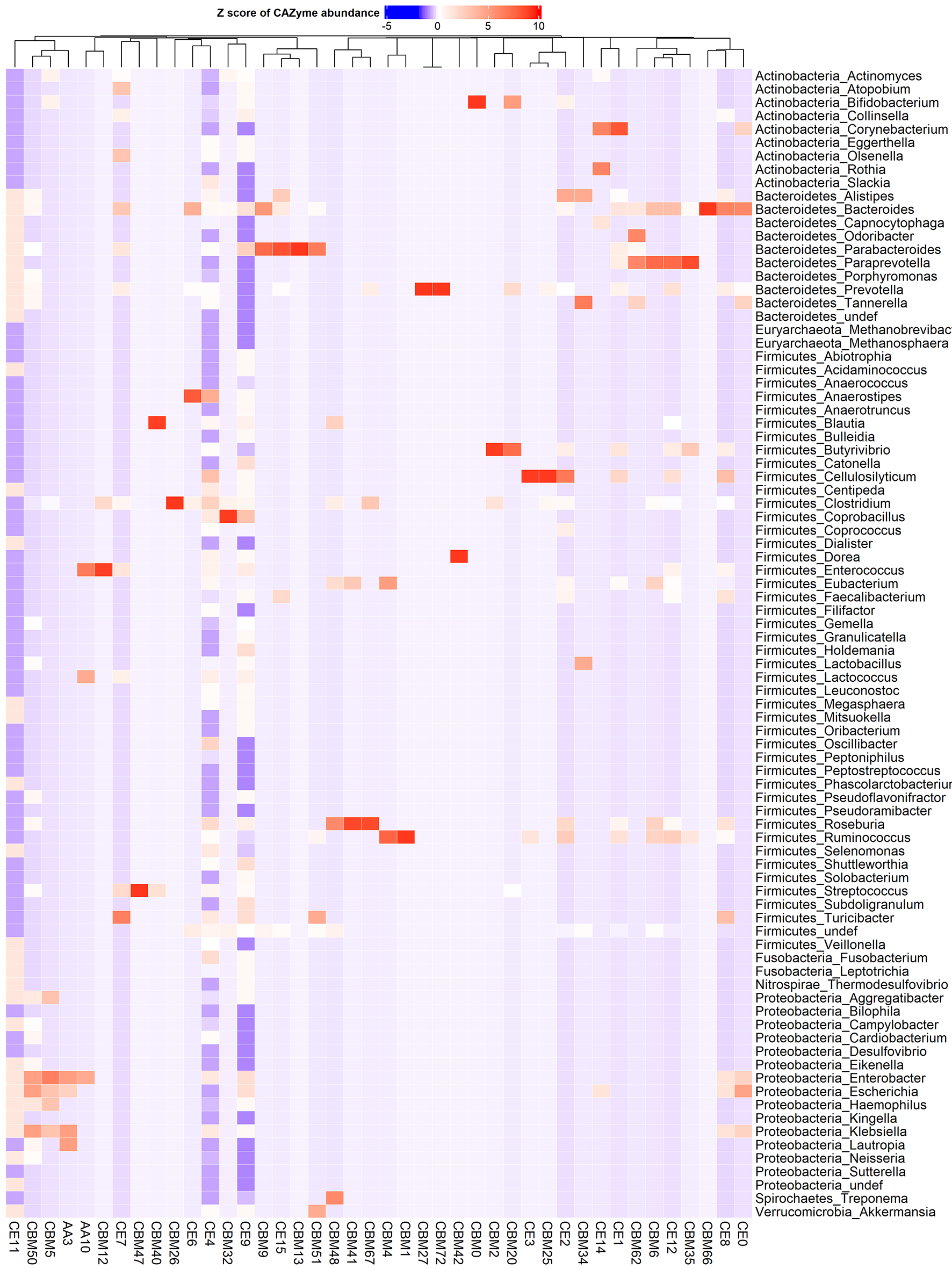

Supplement: Supplementary Figure 2 — Phylum and genus-specific CAZyme family signatures based on Z score standardization. A heatmap of CAZyme family totals annotated from 310 bacterial genomes in 935 metagenomes obtained from the oral (saliva) and gut (fecal) samples of healthy subjects, as well as patients suffering from type 1 diabetes, colorectal cancer, and rheumatoid arthritis. A total of 27 distinct Carbohydrate-Binding Module (CBM), 13 Carbohydrate Esterase (CE), and 2 CAZymes performing Auxiliary Activity functions (AA) from nine phyla and 86 genera are represented. Each genus displays specific CAZyme enrichments covering a broad carbohydrate range, resulting in a diverse symbiotic microbial metabolism. [file Image_2.TIFF]

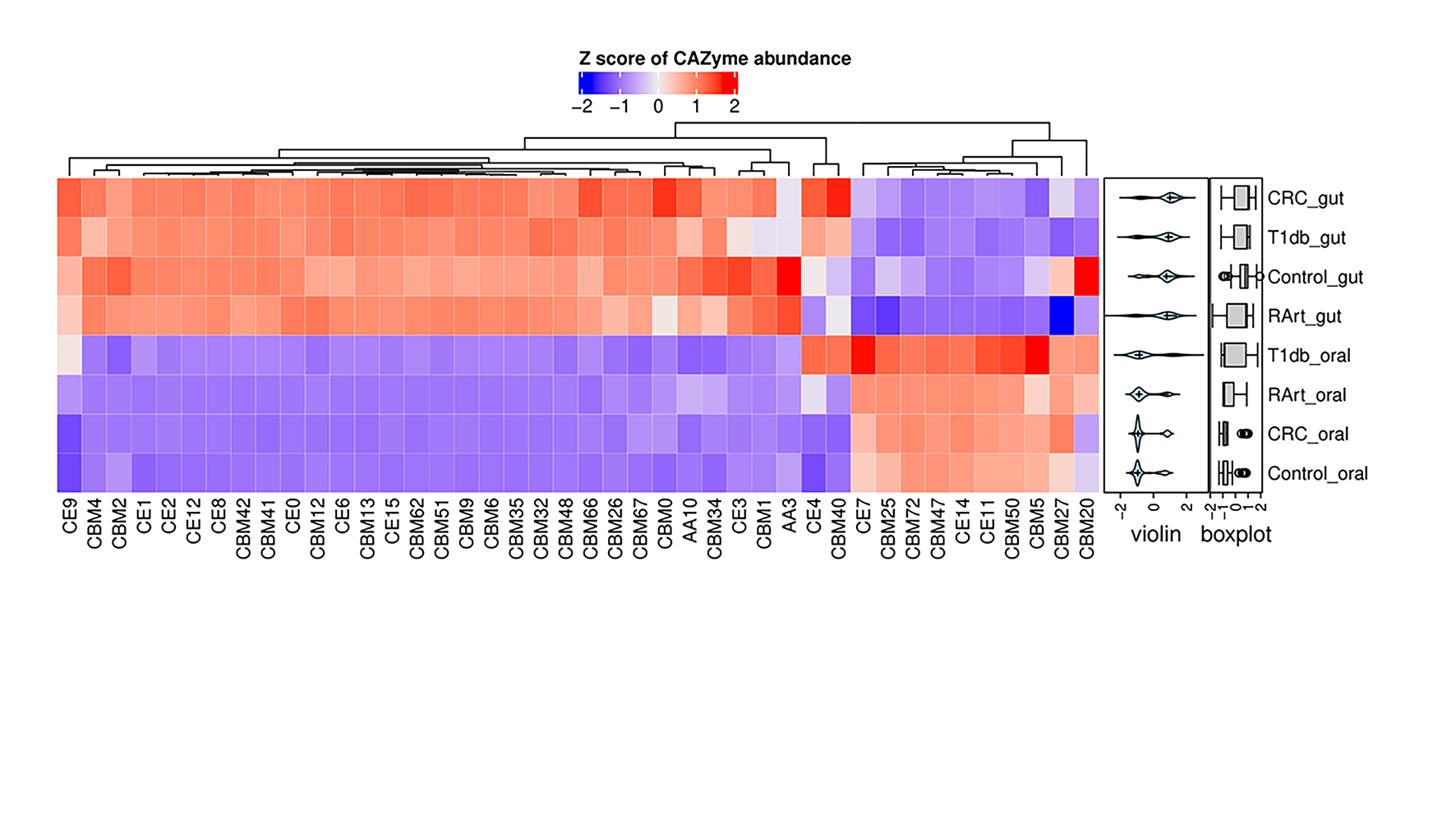

Supplement: Supplementary Figure 3 — A heatmap of CAZyme family distribution in saliva (oral) and fecal (gut) samples of 935 metagenomes derived from healthy controls and patients with type 1 diabetes, colorectal cancer, and rheumatoid arthritis. A total of 27 distinct Carbohydrate-Binding Module (CBM), 13 Carbohydrate Esterase (CE), and 2 CAZymes performing Auxiliary Activity functions (AA) from nine phyla and 86 genera are represented. The oral and gut ecosystems show distinct profiles revealing a more extensive carbohydrate metabolism in the gut. CRC, colorectal cancer; T1db, type 1 diabetes; RArt, rheumatoid arthritis. The legend shows the Z scores of the mean log-transformed CAZyme family abundance. [file Image_3.TIF]

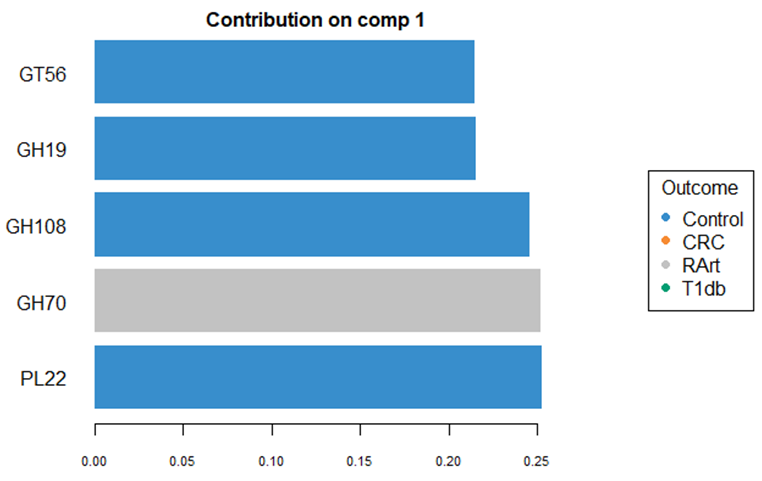

Supplement: Supplementary Figure 4 — Supervised analysis and feature selection with sparse PLS-DA. With the disease status as a categorical variable, the loading weight of the most important CAZyme families selected on component 1 on which the sPLS-DA model was optimal was drawn. Fill colors indicate the disease status in which the CAZyme is most abundant. [file Image_4.TIF]

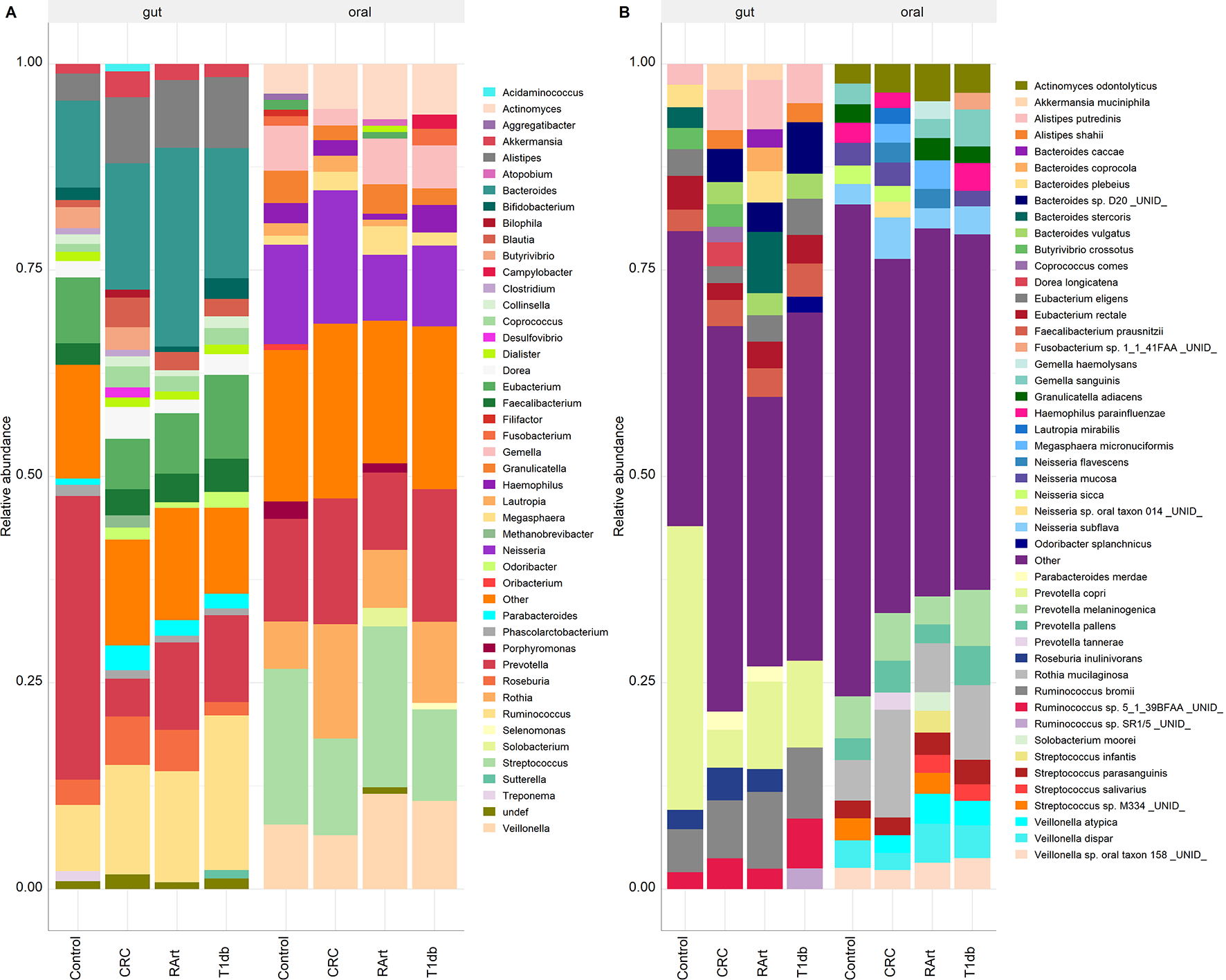

Supplement: Supplementary Figure 5 — Relative abundance of the gut and oral microbial community faceted by disease phenotype at the (A) genus and (B) species level. The abbreviations are disease phenotypes, respectively; CRC, colorectal cancer; RArt, rheumatoid arthritis; T1db, type 1 diabetes. Relative abundance data are adapted from Schmidt et al. (2019). [file Image_5.TIFF]
