# Supplementary material for: Oral and Gut Microbial Carbohydrate-Active Enzymes Landscape in Health and Disease
Source: Front Microbiol. 2021 Dec 10;12:653448. doi: 10.3389/fmicb.2021.653448 (PMC8702856; doi:10.3389/fmicb.2021.653448)
Supplement: Supplementary Table 1 — Sample metagenome mean read depth and number of samples per body site and disease phenotypes. [file Table_1.DOCX]

Table S1. Sample metagenome mean read depth and number of samples per body site and disease phenotypes.

| Disease Phenotype | Body site | Mean read depth | Number  of samples | Reference |
| --- | --- | --- | --- | --- |
| Control | gut | 2.72E+08 | 340 | (Brito et al., 2016; Schmidt et al., 2019; Voigt et al., 2015; Zeller et al., 2014; X. Zhang et al., 2015) |
| Control | oral | 1.06E+08 | 255 |  |
| Colorectal cancer | gut | 1.11E+08 | 26 | (Schmidt et al., 2019; Zeller et al., 2014) |
| Colorectal cancer | oral | 5.16E+07 | 25 |  |
| Diabetes type1 | gut | 9.47E+08 | 41 | (Heintz-Buschart et al., 2016; Schmidt et al., 2019) |
| Diabetes type1 | oral | 5.39E+08 | 38 |  |
| Rheumatoid arthritis | gut | 6.43E+07 | 135 | (X. Zhang et al., 2015) |
| Rheumatoid arthritis | oral | 4.35E+07 | 75 |  |
| Total |  |  | 935 |  |
